# Correction to: MicroRNA-330-3p promotes cell invasion and metastasis in non-small cell lung cancer through GRIA3 by activating MAPK/ERK signaling pathway

**DOI:** 10.1186/s13045-017-0546-4

**Published:** 2018-01-09

**Authors:** Chun-Hua Wei, Gang Wu, Qian Cai, Xi-Can Gao, Fan Tong, Rui Zhou, Rui-Guang Zhang, Ji-Hua Dong, Yu Hu, Xiao-Rong Dong

**Affiliations:** 1 Cancer Center, Union Hospital, Tongji Medical College, Huazhong University of Science and Technology, Wuhan, 430022 China; 2 Medical Research Center, Union Hospital, Tongji Medical College, Huazhong University of Science and Technology, Wuhan, 430022 China; 3 Institute of Hematology, Union Hospital, Tongji Medical College, Huazhong University of Science and Technology, Wuhan, 430022 China

## Correction

The original article [1] contained incorrect affiliations for the authors.

The original article has now been updated to reflect the correct affiliations.
